# Synthetic biology, engineering biology, market expectation

**DOI:** 10.1049/enb.2020.0021

**Published:** 2020-12-02

**Authors:** Lionel Clarke

**Affiliations:** ^1^ BionerG Chester UK

**Keywords:** genetics, cellular biophysics, biology computing, synthetic biology platform, engineering biology, market expectation, research concept, technological platform, commercial products

## Abstract

‘Engineering biology’ is being increasingly adopted as a term by organisations that seek to deliver benefits from ‘synthetic biology’. However, are ‘engineering biology’ and ‘synthetic biology’ different words with the same meaning or do they signal important differences? By observing how these two terms are currently being described and applied in practice, it is possible to differentiate the two whilst also acknowledging significant overlaps and complementarity. Increasing adoption of the term ‘engineering biology’ reflects the maturing of synthetic biology since the early years of this century from a research concept to a technological platform that is facilitating the delivery of commercial products and services. The term ‘synthetic biology’ retains a strong association with its original goal to help make biology engineerable, a challenge that will inevitably continue to stimulate research for decades to come as ever more complex and demanding systems are tackled. In comparison, the term ‘engineering biology’ relates more commonly to the utilisation of the synthetic biology platform alongside other related technologies to deliver effective solutions in response to increasing market challenges and expectations.

The term ‘Engineering Biology’ – the title of this IET Journal – invites us to reconsider our views about the nature of engineering and of biology today. The Collins dictionary includes the following definition: ‘Engineering is the work involved in designing and constructing engines and machinery, or structures such as roads and bridges’ [1]. There are, of course, many other broader definitions to be found in the literature, but the legacy of history generates strong associations between the term ‘engineering’ and the design of inanimate structures, applying mathematics and physics to determine material solutions with predictable outcomes spanning solid, liquid and gaseous phases. Biology is defined in the Collins dictionary as ‘the study of living organisms, including their structure, functioning, evolution, distribution, and interrelationships’ [2]. Likewise, numerous definitions abound, many associating the term ‘biology’ with the idea of the ‘study’ of natural processes. Such definitions do not determine what activity should or should not be but, rather, attempt to reflect in concise terms what is generally understood or practiced. Concise definitions, no matter how carefully crafted, may inevitably fail to reflect the full richness of a vibrant and extensive field of activity, or risk being phrased in such generic terms as to lose the sense of the essential uniqueness that should be associated with a given term.

## 1 Behind the definitions

So what should be made of the term ‘engineering biology’? It draws upon our collective understanding of two very long‐established disciplines, whilst signalling the generation of a radically new and different form of activity. It is not simply describing a cross‐disciplinary application such as the mechanisation of a previously labour‐intensive activity, but a powerful new capability emerging synergistically from the convolution of recent significant developments across a broad range of technologies, including robotics and information and communications technologies (ICTs).

At its core is the field of synthetic biology. Whilst it has been recognised for nearly 70 years that the ‘book of life’ exists within a strand of DNA, it is only very recently that it has become practical and affordable to convert collective understanding into the synthesis of fully functioning organisms. Inspired by the achievements of the Human Genome Project, published in 2003 [3], and drawing upon analogies such as that between the complexity of metabolic circuits and the principles guiding the increasingly automated design of microchip circuitry [4], the field of synthetic biology was born. Modern use of the term ‘synthetic biology’ can be traced most clearly to its first specifically named workshop ‘SB 1.0’ held in June 2004, convened to consider the question ‘what would it take to advance the deliberate and rational engineering of living systems?’ [5]. Despite the very different nature of living biological systems from ‘structures such as roads and bridges’, it was concluded that applying the core engineering principles of standardisation, decoupling, and abstraction could provide a rigorous basis for tackling the challenge [6]. Focusing on the objective of systematic design as a main distinguishing feature of synthetic biology, these general principles have been subsequently re‐expressed in terms of modularisation, characterisation, and standardisation [7].

Adoption of these engineering principles provides a universal framework into which relevant technological advances can be placed and continuously refreshed as improvements are made. For example, standardisation is fundamental to engineering, enabling knowledge to be built and collective progress made through the sharing of unambiguously characterised and reproducible data throughout the community of practitioners. A formal standard for digital biological information BSI PAS 246 was published by the BSI in 2015 [8] and many other standards and protocols continue to be developed, ranging for example from standards for operating language [9] to responsible innovation [10]. ‘Modularisation’ and ‘characterisation’ are also analogous to typical engineering approaches in which devices are built from standard components and systems are built from standard devices [7], but in the case of biological systems, characterisation is determined not simply from the nature of the module but from determining empirically how the target host (cell‐type or strain) responds to its incorporation, such that repeatable behaviour can be predicted. This empirical optimisation stage invokes an implied fourth core principle namely that of learning through doing. The complexity of living systems and the fact that our understanding, whilst advancing rapidly, is still relatively limited, means that the search for optimal solutions in practice requires both the synthesis of multiple options and the application of design–build–test–learn (DBTL) cycles that may be further aided by machine‐learning and artificial intelligence (AI) algorithms. The rigorous, methodical, application of the DBTL approach permits increased predictability and reliability to be achieved.

This structured approach enables increasing automation, an essential facilitator of ever more cost‐effective problem‐solving. In addition, year‐on‐year improvements in cost‐effectiveness and affordability are being accelerated by parallel developments in underpinning tools and techniques, such as CRISP‐Cas9 gene editing and subsequent developments [11], and on‐going advances in contributory technologies in analysis and microfluidics [12] that permit increasingly accurate analyses to be carried out using ever‐smaller volumes of reagents.

Automated high‐throughput ‘biofoundries’ embody the application of these engineering principles. Not only can today's biofoundries carry out genome‐scale engineering [13] or assemble DNA constructs [14] 10 or 20 times faster than an individual in the laboratory, but they also provide the precision and reproducibility of fully automated processes – and can utilise machine learning and AI approaches for optimisation or potentially for discovering entirely novel design options. In a recent ‘stress‐test’ of the biofoundry at SYNBIOCHEM, the Synbio Research Centre at the University of Manchester, a range of 17 industrially relevant material building blocks were successfully designed and constructed within a preset target period of 85 days [15].

The formation of the Global Biofoundries Alliance in 2019 reflects the recognition by the international community of the importance of cooperative initiatives, including the development of standards to promote interoperability, efficiency, and safety. Assembled initially of 15 publicly‐funded biofoundry members from four continents, convened at the Imperial College London in 2018 as part of the SynbiCITE programme [16], membership has already expanded to 27 members [17]. In addition to biofoundries, the formation of ‘cloud laboratories’‐centralised, highly automated laboratories that exploit the increasingly powerful capabilities of computer interfaces and the internet to permit customers to carry out experiments remotely – provides additional options for practitioners to benefit from the engineering principles underpinning synthetic biology [18].

As noted above, definitions serve to summarise fields of activity for ease of communication and clarification. However, for normal operating purposes, they can only reflect, not constrain, the scope of operations, the frontiers of which may continue to progress with time. After more than 15 years of progress, it is possible to review the fitness of definitions applied to ‘synthetic biology’ to date. A variety of definitions of ‘synthetic biology’ emerged from its conceptual beginnings in 2004, of which ‘synthetic biology is the design and engineering of biologically based parts, novel devices and systems as well as the redesign of existing, natural biological systems’ was generated for a synopsis of Synthetic Biology by the Royal Academy of Engineering in 2009 [19] and adopted as a valid working definition for the UK Synthetic Biology Roadmap in 2012 [20]. This particular definition provided a very helpful summary at a time when tools and techniques were still at an early stage of development, and the engineering principles of ‘modularity’ and ‘characterisation’ were being established as an important bridge in mapping the steps required to advance from the design of individual parts towards the design and assembly of more complex organisms. This approach remains today a key first entry point into the world of synthetic biology for tens of thousands of students worldwide who have formed multidisciplinary teams to compete in the annual iGEM competition, drawing upon the ever‐expanding library of ‘parts’ curated by the BioBricks Foundation [21], whilst also promoting the value (and associated culture‐shift) of open‐sourcing previously more prevalent in the world of IT, as represented by the Open Material Transfer Agreement approach [22].

Numerous alternative definitions continued to be generated (reflecting different perspectives, levels of understanding, intended audiences, aspirations, and prejudices) such that, by 2016, the UN Convention on Biological Diversity – needing to establish the nature and scope of synthetic biology as a basis for scientific and technical deliberations under its convention and its protocols – presented a list of more than 30 definition options for consideration via an online forum and its *ad‐hoc* Technical Expert Group (AHTEG). Arising from this exercise, in December 2016 [23] it agreed to adopt an ‘operational definition’ formulated by the AHTEG that ‘synthetic biology is a further development and new dimension of modern biotechnology that combines science, technology and engineering to facilitate and accelerate the understanding, design, redesign, manufacture and/or modification of genetic materials, living organisms and biological systems’.

The term ‘new dimension’ is an important element in this definition. Valid reasons exist to argue that synthetic biology is not simply the next, incremental, step along from the decades of genetic engineering and industrial biotechnology that preceded, but is radically different, potentially transformative, arguably revolutionary. As noted by Kuhn [24], scientific revolutions are not generally obvious (or broadly accepted) at the moment of a particular discovery but tend to become apparent over time as a fresh paradigm stemming from successive reinforcement of the new idea or concept becomes subsequently adopted by the wider community. In the case of synthetic biology, it may be reasonably argued that, in response to the original question, the demonstration that biology is amenable to deliberate and rational engineering, that the application of core engineering principles has helped make biodesign an increasingly accessible and affordable option for non‐specialists to pursue, combined with the cultural shift towards more open, global cooperation that within less than two decades has been embraced by many thousands of practitioners worldwide, does indeed constitute a significant paradigm shift [25].

## 2 Arise, ‘engineering biology’

Whilst the application of engineering principles is firmly embedded in the concept and practice and most definitions of synthetic biology, the alternative title ‘engineering biology’ has been sparingly applied until recently but is now gaining popularity.

An important stakeholder group represented on the UK Synthetic Biology Leadership Council [26] was that of the small–medium enterprise (SME) and start‐up business community. Formed from a group of just eight start‐ups in 2013, under the auspices of The BioIndustry Association, the group has continued to expand, now including SMEs, start‐ups, established companies, and both private and public service providers. Originally termed the Synthetic Biology Advisory Committee, it was decided in 2016 to rename itself as the Engineering Biology Advisory Committee to reflect its primary interest in the development of applications and in promoting the interests of the industrial community [27].

An important contributor to the field of synthetic biology in the US was made by the multi‐university Synthetic Biology Engineering Research Center (SynBERC) during its period of National Science Foundation funding from 2006 to 2016. In 2019, members from SynBERC founded a new programme called the Engineering Biology Research Consortium, building upon its predecessor's technological foundations, but opening its membership to a wider community, not only academic but also industrial, domestic and international. It applies the following definitions for ‘synthetic/engineering biology’: ‘Synthetic biology aims to make biology easier to engineer. Synthetic biology is the convergence of advances in chemistry, biology, computer science, and engineering that enables us to go from idea to product faster, cheaper, and with greater precision than ever before. It can be thought of as a biology‐based ‘toolkit’ that uses abstraction, standardisation, and automated construction to change how we build biological systems and expand the range of possible products’ [28].

In June 2017, the IET published its first volume of this ‘*Engineering Biology*’ journal, noting that ‘synthetic biology is a young and interdisciplinary field of research in the life sciences. It is already revolutionising a number of fields using tools and concepts from physics, engineering and computer science to build new biological systems. *Engineering Biology* is particularly focused on the application of engineering science and practice to the design of biological devices and systems for a wide range of fields and applications. A key aspect is systematic design using the engineering principles of modularity, standardisation and characterisation’ [29].

So is ‘engineering biology’ just an alternative name for ‘synthetic biology’ or is it something different?

One way to approach this question is to consider how the two terms are being used by the community. The Web of Science documents over 8900 research publications using the terms ‘synthetic biology’ or ‘engineering biology’ produced between 2000 and mid‐2020 [30]. Of these, around 98% use the term ‘synthetic biology’ only, a proportion that has actually increased, not decreased, over the past five years. It is clear that the global research community routinely and almost exclusively uses the term ‘synthetic biology’, some adhering very closely to the core engineering principles, others less so. Engineering biology initiatives, as exemplified above, may be considered to focus increasingly on translation for the purpose of developing specific applications and generating solutions to specific market‐based problems. A solutions‐based approach may invoke a range of other technologies as needed, such that synthetic biology may or may not ultimately play a significant role in delivering the final outcome in a particular case, even if it has helped to explore options during the process. Engineering biology embraces synthetic biology's engineering principles, as captured in the definitions noted above, but in principle could also incorporate other technologies as required to achieve a specific commercial target – synthetic biology in this case can be considered a ‘subset’ of engineering biology. It is important that the term synthetic biology remains associated with its more strictly defined goal of making biology engineerable, which has helped drive significant developments by bringing together the biological, ICT, and engineering communities towards a common purpose, efforts that could otherwise become dissipated, stalling the rate of progress. By retaining this clear focus, synthetic biology and engineering biology may be viewed as complementary and overlapping approaches that together help both to generate fresh opportunities and also to deliver effective solutions.

Synthetic/engineering biology has reached the stage of maturity where numerous potentially commercialisable applications are being developed. Several hundred start‐ups and spin‐out companies have already been established, a large proportion of which are located in the US and UK. Private investment into such start‐ups and SMEs was already determined to be well in excess of $12 billion by the end of 2018, increasing at a rate of 30% per year [31]. Products as well as services are now reaching the market. Synthetic biology has extended the possibilities of biodesign to include important but to date a limited number of, mainly prokaryotic organisms. On‐going research, likely to continue throughout the entire 21st century, will be vital to extend its range, both to facilitate more intricate designs within simpler systems and to extend the approach to increasingly complex yet vitally important systems, especially eukaryotes [32].

Engineering biology provides solutions, but what are the problems that need them? Sometimes these cannot be anticipated, but access to leading‐edge technologies can prove valuable when urgent needs arise that are difficult to address using conventional technologies. The very rapid repurposing of the London Biofoundry in response to an urgent need to increase sample testing capacity arising from the recent Covid‐19 pandemic [33] was only possible due to the many previous years of development of the facility. Even before the current Covid‐19 pandemic, public awareness of the consequences at a personal level of global challenges such as climate change had been steadily increasing, engendering mounting recognition of our collective dependency on advancing scientific understanding, whether to help develop more effective therapies or to help ensure long‐term planetary sustainability. Generating effective solutions to such extremely complex issues will require responses at many levels, societal as well as technological, some potentially very disruptive to current practices. Innovative options are increasingly needed to supplement or displace the technological approaches commonly applied today, as fossil‐fuel‐based economies gradually evolve into the bioeconomies of the future. Engineering biology addresses the critical need to identify and translate technological applications arising from cutting‐edge research into safe, effective, and commercialisable responses to increasing market pull. Increasingly urgent market demand for safe and effective solutions, not technology push alone, will determine the rate and extent of uptake.

In 2020, the UK Synthetic Biology Leadership Council has renamed itself as the UK Engineering Biology Leadership Council [34]. Its primary objectives are to provide oversight of the technological cutting‐edge and shifting market need, to address challenges such as those arising from the UK's commitment to achieving net‐zero greenhouse gas emissions by 2050, and to increase the options available to help deliver the UK Bioeconomy and Life Sciences strategies. In this context, it describes engineering biology as ‘an overarching term that incorporates basic research and development (synthetic biology) and industrial translation. Consequently, engineering biology embraces the full range of technologies that must be harnessed to translate biodesign into commercially viable operations and to scale them out to deliver widespread economic prosperity and associated societal benefits’.

## 3 Evolving market expectations

Market demand for better, more effective, solutions are rapidly strengthening as societal awareness of global challenges, ranging from pandemics to climate change, continues to increase. Specific market needs arising as a consequence of current concerns span a broad spectrum, ranging from the demand for more rapid diagnostics and affordable therapies through to lower environmental impact foods, fuels, chemicals, data storage, and more. It is tacitly expected that science and technology will be able to devise and deliver better solutions as and when needed.

Whilst synthetic biology can be viewed as addressing the open‐ended, divergent, phase of discovery and option generation, engineering biology can be considered as addressing the processes of translation and commercialisation in response to market needs, pragmatically combining novel and existing technologies as may be most effective in tackling each specific challenge. Commercialisation represents a more convergent phase, focusing on solving the specific problem being addressed whilst also assimilating all the many factors, including economic, regulatory, and public acceptability – the operational ‘eco‐system’ – that will determine what can and should be done. Every potential application will invoke its own unique set of eco‐system factors engineering biology must accommodate the specifics of individual cases within the context of ever‐heightening market expectations, if the envisaged benefits are to be delivered safely and effectively from this rapidly evolving platform.

Although varying definitions of ‘engineering biology’ will inevitably continue to be generated by different stakeholders for many years, the main drivers for the adoption of this term today relate primarily to the increasing maturity of synthetic biology – with which it is likely to remain intimately linked – as a generator of innovative applications at a time of accelerating market demand driven by fundamental global challenges. The translation of innovative technologies into effective solutions will be determined not by the merits of the technology alone but also by ensuring overall fitness for purpose in addressing the broad range of needs and expectations defined by the market ‘eco‐system’ as a whole.
